# The Late Dr. Armstrong

**Published:** 1831-01-01

**Authors:** 


### XLVIII.

### THE LATE DR. ARMSTRONG.

IT gives us much pleasure to be able to place the following circumstances on record, as proving that, among the members of the medical profession, there is sometimes — perhaps often, manifested a delicacy, a generosity towards each other, for which they are not credited in general. It is only necessary to state that the names of the two physicians alluded to in the following paper, have been communicated to us confidentially, and we can vouch for the high respectability and veracity of the parties.

TO THE EDITOR OF THE MEDICO-CHIRURGICAL REVIEW.

SIR—I read some time since the very judicious and discriminating memoir, given in the Medico-Chirurgical Journal, of the late Dr. Armstrong. The perusal of that article brought to my recollection a circumstance relative to that gentleman's appointment to the Fever Institution, which may not be uninteresting to you and your readers.

At the time when Dr. Armstrong was a candidate for the office of physician to that establishment, his rejection at the Royal College of Physicians took place; and much anxiety was necessarily entertained by him and his friends, as to the effect which this uncomfortable circumstance might have upon his application. It may be remarked, that all appointments at the Fever Institution, were, at that time, made by a Committee of 32 Governors, annually appointed, consisting of a President, six Vice-Presidents, a Treasurer, and 24 other members. Two of the latter were physicians of considerable standing; and Dr. Armstrong called upon one of them, who was well known to him, on the business of the election, a short time before it was to take place, but subsequent to the occurrence, alluded to, at the College. This gentleman stated to Dr. Armstrong, that though he had every disposition to serve him, he felt considerable difficulty as to the mode in which his vote ought in honour to be given, from the unexpected decision of the Examining Board of the College; but that he would give the subject his best attention.

In considering the matter, it appeared to him, on the one hand, that as the College were invested with the power of determining on the qualifications of physicians, and as their examinations were always conducted with courtesy and fairness, their judgement should, in ordinary circumstances, be regarded as decisive of the existence of such qualifications. But, on the other, Dr. Armstrong was not a young man who had just finished his education, but was a physician of many years standing, had been long in considerable eminence and respectable practice in a large provincial town, Sunderland; and was creditably known to the profession by his publications, one of which was on fever, and was therefore calculated to make known, in the best possible way, whatever pretensions he might have, to execute, with ability, the important duties of physician to the Fever Hospital. His deficiencies, too, might be in classical literature; in the nicer parts of anatomy; or in acquaintance with some of those circumstances of professional knowledge which are not frequently called into use, and in which, therefore, a practitioner might, in time, become in some degree rusted. An examiner, however, might feel himself under the necessity of noticing such deficiencies, without reference to the circumstances under which they might occur; while, if he were unfettered by the strict obligation of his official duties, he might look more than he was otherwise authorized to do, to the knowledge required in practice, or to that which was evinced by publications. The inconveniences attaching to a rejection by the College, likewise, were not of formidable extent; the circumstance would be but partially known, and would be the wonder only of a day; while its principal effects would soon be over; for it scarcely ever happens that a second application is not successful. In the case, however, of a failure in the object in question, an opportunity of important advancement might be irrevocably lost, and thus an injury inflicted, of a most serious and lasting character.

On these accounts, but principally from a conscientious belief, that Dr. Armstrong's publications were creditable and useful; and were such as indicated them to be the works of a gentleman of respectable professional attainments, the member of the committee alluded to determined to give his vote for Dr. Armstrong; and on conferring with his brother physician and committee-man on the subject, he had the gratification to find him of the same opinion. The election was therefore unanimously in favour of Dr. Armstrong.

I am desirous of mentioning these circumstances, because it has been stated, that Dr. Armstrong had severe and constant opposition from his professional brethren in the metropolis, who have often been represented as envious of his reputation and merit. He owed, however, his appointment to the Fever Hospital, in an eminent degree, to the manly and independent feelings of his medical brethren of the committee; for it is difficult to conceive, that any board, executing before the world a public trust, would, against the opinion of the only two professional men of their body, and in the face of the College rejection, have appointed any one to the most important and responsible situation of a medical establishment. It can hardly therefore be conceived, that one gentleman could receive a more signal mark of kindness from others, than Dr. Armstrong did on the occasion in question. And the value of the obligation may sufficiently be appreciated by those who consider, on the one hand, how much the loss of a position which was looked to for professional success, would have interfered with advancement in the metropolis; and on the other, how difficult it must have been, for gentlemen of delicate professional feelings to appear to set themselves (though in the modified way which has been stated) against the opinion of a body for which they felt much respect, and to which they owed a species of professional allegiance.

I have the honor to be,
Sir,
Your most obedient servant,
*Nov. 26th*, 1830. X. Y. Z.

